# Prerequisites for implementing physical activity on prescription for children with obesity in paediatric health care: A cross-sectional survey

**DOI:** 10.3389/frhs.2022.1102328

**Published:** 2023-01-30

**Authors:** Charlotte Boman, Susanne Bernhardsson, Katarina Lauruschkus, Stefan Lundqvist, Karin Melin

**Affiliations:** ^1^ Centre for Physical Activity, Region Västra Götaland, Gothenburg, Sweden; ^2^ Unit of Physiotherapy, Department of Health and Rehabilitation, Institute of Neuroscience and Physiology, Sahlgrenska Academy, University of Gothenburg, Gothenburg, Sweden; ^3^ Research, Education, Development and Innovation, Primary Health Care, Region Västra Götaland, Gothenburg, Sweden; ^4^ Faculty of Medicine, Institution of Health Sciences, Lund University, Lund, Sweden; ^5^ Department of Habilitation, Committee on Psychiatry, Habilitation and Technical Aids, Region Skåne, Lund, Sweden; ^6^ Institute of Health and Care Sciences, Sahlgrenska Academy, University of Gothenburg, Gothenburg, Sweden; ^7^ Department of Child and Adolescent Psychiatry, Sahlgrenska University Hospital, Region Västra Götaland, Gothenburg, Sweden

**Keywords:** obesity, physical activity on prescription, children, feasability, implementation, determinants, normalization process theory (NPT), survey

## Abstract

**Background:**

Physical inactivity is a main driver of childhood obesity that tracks into adulthood, making it crucial to address early in life. Swedish physical activity on prescription (PAP) is an effective intervention for increasing physical activity levels in adults and is being implemented in primary care in Sweden. Before implementing PAP for children, both intervention effectiveness and implementation prerequisites need to be examined. Framed by the Normalization Process Theory (NPT) domains, this study aimed to investigate perceptions of PAP amongst paediatric staff and managers working with children with obesity, as well as acceptability, appropriateness, feasibility, and barriers and facilitators for implementing PAP in paediatric health care.

**Methods:**

Staff and managers in 28 paediatric outpatient clinics in western Sweden were surveyed using validated implementation instruments and open-ended questions. Data were analysed using Mann–Whitney U tests and Kruskal–Wallis tests. Qualitative data were categorised into NPT domains.

**Results:**

The survey response rate was 54% (125/229). Most respondents (82%) reported PAP to be familiar and many (56%) perceived it as a normal part of work; nurses and physiotherapists to a greater extent (*p* < 0.001). This was anticipated to increase in the future (82%), especially amongst those with the longest work experience (*p* = 0.012). Respondents reported seeing the potential value in their work with PAP (77%), being open to working in new ways to use PAP (94%), and having confidence in their colleagues' ability to use PAP (77%). Barriers and facilitators were found in all the NPT domains, mainly collective action and reflexive monitoring, where, for example, inadequacies of education, resources, and research on PAP for children were reported as barriers. Most respondents agreed that PAP was acceptable, appropriate, and feasible (71% to 88%).

**Conclusions:**

PAP is familiar and perceived as an acceptable, appropriate, and feasible intervention, and by many viewed as a normal part of clinical routines in paediatric outpatient clinics in western Sweden, especially by physiotherapists and nurses. Barriers and faciliators are mainly related to collective action and reflexive monitoring. The wide acceptance demonstrates receptiveness to PAP as an intervention to promote an active lifestyle for children with obesity.

## Introduction

Childhood obesity has increased dramatically in recent decades and prevalence remains high in many countries (1, 2), making it an urgent public health concern. The prevalence of obesity in European children aged 5–9 years was 11.4% in 2016 (3). In Sweden, 6% of children aged 6–9 had obesity in 2019, an increase by 4% since 2016 (4). Additionally, the COVID-19 pandemic has driven weight gain amongst children (5–9), caused for example by decreased physical activity, increased screen time, and increased dietary intake (10). Obesity is considered a complex multifactorial condition (11), which tracks into adulthood and is associated with cardiometabolic and psychosocial comorbidity as well as premature mortality (12–15). One of the main behavioural drivers and an important risk factor, is physical inactivity (16, 17), making it critical to address this issue early in life.

For children who are overweight or obese, studies have shown positive effects of physical activity on weight-related outcomes, e.g., body fat and insulin resistance (18, 19), while evidence for interventions to increase children's overall physical activity levels remains inconsistent (20, 21). However, research highlights that although there is evidence for physical activity interventions, implementation strategies to translate evidence-based results into practice are lacking (22, 23). In paediatric health care, behaviour-changing interventions are commonly used with the aim to improve dietary intake, increase physical activity, and reduce sedentary time (16). Physical activity on prescription (PAP) is one such intervention that is being implemented in many countries, including Sweden, to promote lifestyle change in the form of increasing physical activity (24) and decreasing sedentary time (25). The Swedish PAP intervention comprises three core components: a person-centred dialogue, individually tailored activity recommendation with a written prescription, and a structured follow-up (26).

Studies of PAP have shown effectiveness in adults, including patients with overweight or obesity, measured as increased physical activity levels (27), but for children there is a paucity of studies. One study (28) showed PAP to be both feasible and increasing physical activity levels amongst children with cerebral palsy, and one study (29) showed effects on BMI scores in children with obesity after a web-based intervention of which PAP was one component. The Swedish National Board of Health and Welfare's National Guidelines for Methods of Preventing Disease posit PAP as an evidence-based practice targeting adults (30). Because an inactive lifestyle amongst Swedish children and youth is a common health concern (31), several regions in Sweden have started to use PAP for physically inactive children. As part of a combined lifestyle treatment, PAP might be a potentially behaviour-changing and structured intervention for children with obesity, in accordance with the description of requirements and needs in the national guidelines for treatment of childhood obesity in Sweden (32).

Several barriers and facilitators for implementation of health promoting interventions have been identified. A recent review showed that implementation support strategies, such as educational materials and meetings, opinion leaders, small incentives or grants, and tailored interventions may improve implementation of programmes to prevent obesity and promote physical activity for young children (23). For adults, identified barriers for implementing PAP include practitioners' lack of knowledge about the intervention and lack of organisational support (33–36). Reports including paediatric contexts also identified lack of time, lack of evidence for PAP for children, and limited collaboration with activity organisers as barriers (37, 38). Facilitators include affirmative attitudes amongst practitioners' and central and local supporting structures. However, no study has investigated the prerequisites amongst staff and managers for implementing PAP for children with obesity in paediatric health care.

Identifying implementation determinants before implementing a new intervention is crucial for implementation success. Especially in the highly complex healthcare context (39), implementing new interventions can be challenging. It is also important to understand what works and does not work in the implementation process, for which using a theory is recommended (40, 41). The Normalization Process Theory (NPT), especially developed for use in health care, was designed to help us understand how complex interventions become implemented in routine healthcare practice (39). This theory is concerned with explaining the work people do during the implementation process, and comprises four core constructs, or domains (42). The constructs/domains can be described as a set of mechanisms that energise and shape implementation processes, with a focus on how an intervention can become part of everyday practice (43), making them relevant to assess before implementing a new intervention.

Other implementation determinants that are important to assess before implementing a new intervention are the acceptability, appropriateness, and feasibility of the intervention; three determinants often used during early-stage implementation and seen as leading indicators of implementation success (44). There is a lack of knowledge about whether PAP is perceived as a suitable intervention for children with obesity. To address this knowledge gap, it is important to investigate the prerequisites, barriers and facilitators amongst staff and managers for implementing PAP for childhood obesity in paediatric health care. This knowledge is highly warranted before the intervention is implemented more widely.

The aims of this study were to examine (1) how staff and managers perceive PAP for children with obesity in terms of the NPT domains coherence, cognitive participation, collective action, and reflexive monitoring; (2) what barriers and facilitators they report for working with PAP for children with obesity; and (3) how they perceive acceptability, appropriateness, and feasibility of PAP for children with obesity.

## Methods

### Study design and setting

The study design was a cross-sectional survey, guided by the NPT and collecting quantitative and qualitative data using a web-based questionnaire. Findings are reported, when applicable, according to the STROBE checklist (45).

The study was conducted in the paediatric healthcare organisations in Region Västra Götaland, Sweden, comprising 26 clinics, and four rehabilitation clinics providing healthcare services for children with obesity. The organisations all cater to children with obesity and offer specialist health services. Region Västra Götaland is Sweden's second largest county council, providing healthcare services to approximately 1.7 million residents in western Sweden. One major city, Gothenburg, is located in the region, while the rest of the region comprises three smaller cities, medium-sized towns, and rural areas located in four regional areas (Table 1). In Gothenburg, PAP has already been introduced amongst healthcare professionals, through for example education, tutoring, networking, and PAP clinics supporting families whose children have been prescribed physical activity.

**Table 1:** Participating clinics by geographic location (*n* = 30).

Gothenburg	Regional area			
	Södra Bohuslän	Fyrbodal	Skaraborg	Södra Älvsborg
POC Frölunda	POC Kungälv	POC Dalsland	POC Lidköping	POC Alingsås
POC Hisingen	POC Mölndal	POC Lysekil	POC Mariestad	POC Lerum
POC Kungshöjd	POC Mölnlycke	POC NÄL	POC SkaS	POC Skene
POC Öckerö	POC Partille	POC Strömstad	POC Skövde	POC Ulricehamn
Obesity centre at Queen Silvia's Children's Hospital	POC Stenungssund			POC Viskan
Primary care rehabilitation clinic, Angered	POC Tjörn			Primary care rehabilitation clinic, Lerum
Primary care rehabilitation clinic, Gamlestaden				Primary care rehabilitation clinic, Sörhaga
Specialist centre for children and youth, Angered				
Specialist centre for children and youth, Gamlestaden				

POC, paediatric outpatient clinic.

### Participants

The inclusion criterium for participating in the survey was to be either staff or manager at a paediatric healthcare clinic or a rehabilitation clinic providing outsourced rehabilitation services for children with obesity, in Region Västra Götaland. No prior experience of working with PAP was required. Approximately 240 eligible participants were identified with the assistance of managers and administrative staff. The heads of departments approved the clinics' participation in the study; all 30 clinics accepted the invitation to participate.

### Data collection and outcomes

All eligible participants were invited to answer a web-based questionnaire comprising four validated instruments measuring implementation outcomes. The questionnaire was distributed *via* e-mail during a three-week period in February and March 2021. To increase response rate and reduce the risk of non-response bias, three reminders at one-week intervals were sent.

In this study, the NPT was used to investigate and understand the collective work with PAP for children with obesity at the paediatric healthcare clinics. To assess the implementation process from the perspective of staff directly involved in the work of implementing PAP and their managers, the Normalization MeAsure Development tool (NoMAD) (42) was used. This instrument was specifically developed for implementation in healthcare contexts (42) and can be applied at any stage of an implementation process (46). It is adaptable to different interventions and settings, and can be combined with other measurements focusing on other dimensions of implementation (42).

The NoMAD instrument consists of 23 items, of which three general questions are indicators of normalisation answered on 11-point Likert-type scales ranging from “still feels very new” to “feels completely familiar” for item 1 and from “not at all” to “completely” for items 2 and 3 (46). Twenty items target the four core NPT domains: (1) coherence, that is the “sense-making” work people do to initiate a new intervention or practice; (2) cognitive participation, described as the relational work around the practice; (3) collective action, the work to perform/operationalise the practice; and (4) reflexive monitoring, the appraisal work to understand the new practice (42). Each item has two options, A and B. Option A is answered on 5-point Likert-type scales with response options “strongly agree”, “agree”, “neither agree nor disagree”, “disagree”, and “strongly disagree”. Option B is applicable only to those who found no relevance in answering option A (46).

In this study the validated Swedish version S-NoMAD (47) was used. The word “intervention” was replaced by “PAP for children with obesity” or just “PAP”. Although the instrument was developed for healthcare professionals directly involved in the intervention, we wanted to address the perspectives of both staff and managers. Some items were slightly modified by adding wording addressing managers, e.g., “Do you feel PAP is currently a normal part of your work/area of responsibility?” and “Management supports/I as a manager support PAP for children with obesity”.

To supplement NoMAD, the implementation determinants acceptability, appropriateness, and feasibility of implementing PAP in paediatric health care were assessed, adding the staffs’ and managers' perceptions and attitudes towards PAP. Acceptability is defined as the perception amongst stakeholders that a given treatment, service, practice, or innovation is agreeable, palatable, or satisfactory (44). Appropriateness is the perceived fit, relevance, or compatibility of the innovation or evidence-based practice for a given practice setting, provider, or consumer; and/or perceived fit of the innovation to address a particular issue or problem. Feasibility is the extent to which a new treatment, or an innovation, can be successfully used or carried out within a given agency or setting (44). These outcomes were measured with the Acceptability of Intervention Measure (AIM), Intervention Appropriateness Measure (IAM), and Feasibility of Intervention Measure (FIM) (48). All three are validated instruments with the purpose of assessing the fit and match of a practice or intervention to a given context, targeting different criteria (48). The measures comprise four items each, answered on 5-point ordinal scales with response options “strongly agree”, “agree”, “neither agree nor disagree”, “disagree”, and “strongly disagree”. The instruments were translated and cross-culturally adapted into Swedish, adapted to children with obesity, and validated (49).

In addition, we collected demographic data and data on PAP experience and prescribing frequency. Two open-ended questions explored barriers and facilitators, in which the respondents were given the opportunity to describe their own experiences and thoughts regarding determinants for implementing PAP.

### Data analysis

The quantitative variables are presented descriptively using frequencies and percentages and medians and interquartile ranges. To facilitate future comparisons with other studies, means and standard deviations (SD) are also presented. The respondents' practice location was categorised into Gothenburg and other regional areas of the Region Västra Götaland (Table 1). Work experience in the organisation was categorised into (1), *<*2 *years;* (2), 2–5 *years;* (3), 6–10 *years; and* (4), *>*10 *years.* Professions were categorised into six groups: (1), *nurse;* (2), *physician;* (3), *dietician;* (4), *physiotherapist*; (5), *manager* and (6), *other*. Number of years working with/taking decisions about PAP was categorised into (1), *<*3 *years*; (2), 3–5 years; and (3), >5 *years or longer.* Missing data analyses were performed using chi-square tests to examine proportion of managers and practice location amongst non-responders vs. responders.

To facilitate presentation and interpretation, response categories were merged into fewer categories. Responses to the three general items were coded as: 0–4 = *not familiar* and 5–10 =* familiar* for item 1; 0–4 = *not a normal part of work* and 5–10 = *a normal part of work* for item 2; and 0–4 = *it will not become a normal part of work* and 5–10 = *it will become a normal part of work* for item 3. For the NoMAD items, the *disagree/strongly disagree* response categories were merged into *disagree,* and the *strongly agree/agree* response categories were merged into *agree*. Item 3.2 was reverse-scored due to its negative wording. One item (2.2) was not analysed since it was accidentally removed from the questionnaire. For the AIM/IAM/FIM suite of instruments, the response categories *completely disagree/disagree* were merged into *disagree* and *agree/completely agree* were merged into *agree*.

Comparative analyses of participants from the Gothenburg clinics in which PAP has already been introduced vs. clinics in the rest of the region were performed using chi-square tests for the dichotomised general questions. Because the assumptions of the chi-square tests were not met for the NOMAD and AIM/IAM/FIM items, we performed Mann–Whitney *U* tests using the original 5-point scales. Differences between years of work experience in the organisation and between professions in all variables were performed using Kruskal–Wallis tests with pairwise comparisons, applying Bonferroni correction. For variables where there were significant differences in the main Kruskal-Wallis test, we only report significant differences in the pairwise comparisons. Because age and work experience correlated, no comparisons were made between age groups. A *p*-value of ≤0.05 was considered statistically significant. All cases for which all items in at least one instrument were completed, were included in the analyses.

Internal consistency of the NoMAD items was acceptable for coherence (Cronbach's *α* = 0.748), and questionable for cognitive participation (*α* = 0.600), collective action (*α* = 0.638), and reflexive monitoring (*α* = 0.687). For the the AIM/IAM/FIM measures, internal consistency was excellent for acceptability and appropriateness (Cronbach's *α* = 0.924 and 0.943, respectively) and good for feasibility (*α* = 0.892). Quantitative data were analysed using IBM SPSS, version 28 (IBM Corp, Armonk, NY).

The barriers and facilitators described in free text answers to the open-ended questions were coded and sorted into categories corresponding to the NPT domains. This was done in an iterative process by the first author together with two physiotherapist colleagues with experience of working with PAP for children.

## Results

A total of 229 healthcare professionals (of whom 30 managers) were invited to participate in the survey, and 125 responded (response rate 54.5%). Of the 104 non-responders, 18 were managers. Missing data analysis showed no significant differences between responders and non-responders related to the proportion of managers or practice location in the Gothenburg area vs. other regional areas. Item-level missing values ranged from 7 to 12 (5.6%–9.6%) for S-NoMAD and from 0 to 8 (0.0%–6.4%) for AIM, IAM, and FIM. Mean age of the respondents was 48.2 years (SD 9.6). Respondent characteristics are presented in Table 2.

**Table 2:** Respondent demographic characteristics (*n* = 125).

Characteristic	*n* (%)
**Age (years)**	
<30	4 (3.2)
30–39	21 (16.8)
40–49	44 (35.2)
50–59	37 (29.6)
>59	19 (15.2)
**Work experience in the organisation (years)**	
<2	26 (20.8)
2–5	42 (33.6)
6–10	27 (21.6)
>10	30 (24.0)
**Profession**	
Nurse, including paediatric nurse	43 (34.4)
Physician, including paediatrician	32 (25.6)
Dietician	13 (10.4)
Physiotherapist	9 (7.2)
Manager	12 (9.6)
Other^a^	16 (12.8)
**Role in relation to PAP**	
Works with PAP	68 (54.4)
Is aware of PAP but does not work with it	56 (44.8)
Is not aware of PAP	1 (0.8)
**Experience of working with PAP (years)**	
<3	62 (49.6)
3–5	31 (24.8)
>5	32 (25.6)
**Frequency of prescribing PAP**	
Prescribers	64 (51.2)
Daily	2 (1.6)
Once per week	10 (8.0)
Once per month	27 (21.6)
Once per year	25 (20.0)
Non-prescribers	61 (48.8)

PAP, physical activity on prescription.

^a^ Other = psychologist, social counsellor, occupational therapist, and nursing assistant.

### General questions about PAP

A majority of the respondents (81.1%) reported being familiar with PAP (Table 3). A higher proportion of respondents in the Gothenburg area reported being familiar with PAP than those in regional areas (90.0% vs. 70.6%, *χ*^2^ = 6.772, *p* = 0.009). Physiotherapists reported familiarity with PAP to a greater extent than “other” professions (Mdn 9 vs. Mdn 5, U = 55, *p* = 0.025). Fifty-six percent described PAP as currently being a normal part of their work; a higher proportion of respondents from the Gothenburg area reported this than those in the regional areas (70.0% vs. 40.7%, *χ*^2^ = 9.882, *p* = 0.002). Nurses reported feeling PAP was a normal part of their work to a greater extent than “other” professions (Mdn 6.5 vs. Mdn 0.5, *U* = 38, *p* < 0.001), as did physiotherapists (Mdn 9 vs. Mdn 0.5, *U* = 53, *p* < 0.001). A majority (82.0%) reported believing that PAP will become a normal part of their work. Respondents with >10 years of work experience in the organisation reported this belief to a greater extent than those with 2–5 years' experience (Mdn 9 vs. Mdn 6, *U* = 23, *p* = 0.012).

**Table 3:** General questions about physical activity on prescription.

Items	*N* (missing)	0–4 1. Not familiar 2. Currently not a normal part of work 3. Will not become a normal part of work *n* (%)	5–10 1. Familiar 2. Currently a normal part of work 3. Will become a normal part of work *n* (%)	Median (IQR)^a^	Mean (SD)^a^
1. When you use PAP, how familiar does it feel?	111 (14)	21 (18.9)	90 (81.1)	7 (5–9)	6.53 (2.61)
2. Do you feel PAP is currently a normal part of your work*/area of responsibility?*	114 (11)	50 (43.9)	64 (56.1)	5 (2–8)	4.99 (3.40)
3. Do you feel PAP will become a normal part of your work*/area of responsibility?*	89 (36)	16 (18.0)	73 (82.0)	7 (6–9)	7.08 (2.62)

Text in italic font are adjustments made so that the item would be answerable also by managers.

^a^ Medians and means are calculated on the original 11-point scale.

IOR, interquartile range; PAP, physical activity on prescription; SD, standard deviation.

### Coherence

Most respondents (67.9%) agreed that they could distinguish between PAP and their usual ways of working, and 56.1% reported that they have a shared understanding of its purpose (Table 4). Respondents in the Gothenburg area agreed to a greater extent than those in regional areas to having a shared understanding of PAP (Mdn 4 vs. Mdn 3, *U* = 1170, *p* = 0.005) and of how the intervention affects the nature of their work (Mdn 4 vs. Mdn 3, *U* = 1104, *p* = 0.017) (Table 5). About three quarters of the respondents (76.6%) agreed on the potential value of PAP. No differences were seen related to work experience in the organisation or profession in this domain. Option B responses were selected by 3–8 respondents (2.6% to 6.8%).

**Table 4:** Responses to NoMAD by Normalization Process Theory domain.

Domain	Option A						
	*N* (missing)	*n* Option A	Agree *n* (%)	Neutral *n* (%)	Disagree *n* (%)	Median (IQR)^a^	Mean (SD)^a^
**Coherence**							
I can see how PAP differs from usual ways of working	117 (8)	112	76 (67.9)	28 (25.0)	8 (7.1)	4 (3–4)	3.8 (0.87)
Staff in this organisation have a shared understanding of the purpose of PAP	117 (8)	114	64 (56.1)	45 (39.5)	5 (4.4)	4 (3–4)	3.6 (0.77)
I understand how PAP affects the nature of my own*/my staff's* work	114 (11)	108	52 (48.1)	50 (46.3)	6 (5.6)	3 (3–4)	3.5 (0.72)
I can see the potential value of PAP for my work	115 (10)	107	82 (76.6)	22 (20.6)	3 (2.8)	4 (4–4)	3.92 (0.71)
**Cognitive participation**							
There are key people who drive PAP forward and get others involved	115 (10)	108	51 (47.2)	42 (38.9)	15 (13.9)	3 (3–4)	3.4 (1.0)
I’m open to working with colleagues/ staff in new ways to use PAP	117 (8)	109	103 (94.5)	6 (5.5)	0 (0.0)	4 (4–5)	4.4 (0.59)
I will continue to support PAP	115 (10)	112	96 (85.7)	16 (14.3)	0 (0.0)	4 (4–5)	4.3 (0.7)
**Collective action**							
I can easily integrate*/take decisions about PAP into my existing work*	116 (9)	109	63 (57.8)	38 (34.9)	8 (7.3)	4 (3–4)	3.7 (0.88)
PAP disrupts working relationships^b^	118 (7)	110	2 (1.8)	15 (13.6)	93 (84.5)	4 (4–5)	4.24 (0.79)
I have confidence in *my colleagues/staff's* ability to use PAP	118 (7)	114	88 (77.2)	24 (21.0)	2 (1.8)	3 (1–2)	4.04 (0.75)
Work is assigned to those with skills appropriate to PAP	117 (8)	107	60 (56.0)	40 (37.4)	7 (6.5)	3 (2–3)	3.6 (0.75)
Sufficient training is provided to enable staff/managers to implement PAP	116 (9)	100	26 (26.0)	40 (40.0)	34 (34.0)	4 (2–4)	2.9 (0.93)
Sufficient resources are available to support PAP	118 (7)	108	29 (26.9)	48 (44.4)	31 (28.7)	4 (2–4)	2.93 (1.02)
Management*/I as a manager* adequately supports PAP	113 (12)	90	46 (51.1)	39 (43.3)	5 (5.6)	3 (2–3)	3.6 (0.78)
**Reflexive monitoring**							
I am aware of reports about the effects of PAP	116 (9)	113	42 (37.1)	37 (32.7)	34 (30.1)	3 (2–4)	3.08 (1.06)
The staff agree that PAP is worthwhile	17 (8)	113	68 (60.2)	40 (35.4)	5 (4.4)	2 (2–3)	3.7 (0.75)
I value the effects that PAP has had on my work	116 (9)	99	48 (48.5)	46 (46.5)	5 (5.1)	3 (2–3)	3.51 (0.75)
Feedback about PAP can be used to improve it in the future	115 (10)	109	89 (81.2)	18 (16.5)	2 (1.8)	2 (2–2)	4.02 (0.73)
I/*the staff* can modify how I/*they* work with PAP	117 (8)	104	63 (60.6)	36 (34.6)	5 (4.8)	2 (2–3)	3.63 (0.78)

IQR, interquartile range; SD, standard deviation.

^a^ Medians and means are calculated on the original 5-point scale.

^b^ Item reverse scored. In the survey most of the items were formulated as *PAP for children with obesity.* Text in italic font are adjustments made so that the item would be answerable also by managers.

**Table 5:** NoMAD responses by Normalization Process Theory domains and practice location.

Areas	Gothenburg (*n* = 66)			Regional (*n* = 59)			*p* value*
	*n* (missing)	Median (Q1;Q3)	Mean	*n* (missing)	Median (Q1;Q3)	Mean	
**Coherence**							
I can see how PAP differs from usual ways of working	56 (10)	4 (3;4)	3.88	56 (3)	4 (3;4)	3.73	0.407
Staff in this organisation have a shared understanding of the purpose of PAP	57 (9)	4 (3;4)	3.79	57 (2)	3 (3;4)	3.40	**0** **.** **005**
I understand how PAP affects the nature of my own/*my staff's* work	56 (10)	4 (3;4)	3.64	52 (7)	3 (3;4)	3.29	**0** **.** **017**
I can see the potential value of PAP for my work	55 (11)	4 (4;4)	3.98	52 (7)	4 (3;4)	3.87	0.544
**Cognitive participation**							
There are key people who drive PAP forward and get others involved	52 (14)	4 (3;4)	3.63	56 (3)	3 (3;4)	3.09	**0** **.** **003**
I’m open to working with colleagues/*staff* in new ways to use PAP	54 (12)	4 (4;5)	4.33	55 (4)	4 (4;5)	4.44	0.325
I will continue to support PAP	57 (9)	4 (4;5)	4.30	55 (4)	4 (4;5)	4.24	0.675
**Collective action**							
I can easily integrate/*take decisions* about PAP into my existing work	55 (11)	4 (3;4)	3.73	54 (5)	4 (3;4)	3.63	0.488
PAP disrupts working relationships^a^	56 (10)	2 (1;2)	1.71	54 (5)	2 (1;2)	1.81	0.680
I have confidence in my colleagues/*staff's* ability to use PAP	58 (8)	4 (4;5)	4.12	56 (3)	4 (3;5)	3.95	0.296
Work is assigned to those with skills appropriate to PAP	54 (12)	4 (3;4)	3.74	53 (6)	3 (3;4)	3.43	**0** **.** **032**
Sufficient training is provided to enable staff/*managers* to implement PAP	49 (17)	3 (3;4)	3.24	51 (8)	2 (2;3)	2.55	**<0** **.** **001**
Sufficient resources are available to support PAP	55 (11)	3 (3;4)	3.13	53 (6)	3 (2;3)	2.74	**0** **.** **029**
Management/*I as a manager* adequately supports PAP	41 (25)	4 (3;4)	3.73	49 (10)	3 (3;4)	3.45	0.059
**Reflexive monitoring**							
I am aware of reports about the effects of PAP	56 (10)	3 (2;4)	3.11	57 (2)	3 (2;4)	3.05	0.995
The staff agree that PAP is worthwhile	56 (10)	4 (3;4)	3.73	57 (2)	4 (3;4)	3.58	0.406
I value the effects that PAP has had on my work	53 (13)	4 (3;4)	3.68	46 (13)	3 (3;4)	3.30	**0** **.** **011**
Feedback about PAP can be used to improve it in the future	56 (10)	4 (4;5)	4.05	53 (6)	4 (4;4)	4.00	0.761
I/*the staff* can modify how I/*they* work with PAP	54 (12)	4 (3;4)	3.63	50 (9)	4 (3;4)	3.64	0.864

^a^ Item reverse scored. *PAP* Physical activity on prescription. In the survey most of the items were formulated as *PAP for children with obesity.*

*p-values are derived from Mann-Whitney U tests of differences between practice locations.

A barrier for using PAP described in the open-ended questions was the respondents' experiences of not knowing the PAP intervention well enough and working with single components alone, particularly the written prescription for physical activity. The opposite, a comprehension of the PAP intervention and considering and including all of its components, was described as a facilitator. Statements like “I consider it important that PAP is well supported by a good assessment so it will be at the right level, for example goal setting, activity, duration, and that the patient is motivated. If not, then it might just be ‘another piece of paper’ for the individual.” were typical.

### Cognitive participation

Almost half (47.2%) agreed that there are key people who drive PAP forward and get others involved. Respondents in Gothenburg agreed to a greater extent than those in regional areas that there are key people driving PAP forward and involving others (Mdn 4 vs. Mdn 3, *U* = 1000, *p* = 0.003). Most reported being open to working with colleagues in new ways to use PAP (94.5%) and agreed to continuing to support PAP (85.7%). No differences were seen related to work experience or profession in this domain. Option B responses were selected by 1–8 respondents (0.9% to 6.8%).

A reported barrier in this domain for using PAP was the absence of physiotherapists at the clinics and the perceived uncoordinated pathways to healthcare units offering PAP support. Facilitators for using PAP were colleagues being supportive of PAP and successful healthcare collaboration. Statements like “In my clinic we have divided the tasks between us a little. However, I could prescribe PAP more often, but mostly it's done by my colleague who is a nurse.” were reported.

### Collective action

Over half of the respondents (57.8%) agreed they can easily integrate PAP into their existing work and only 1.8% agreed that PAP disrupts working relationships. A majority (77.2%) reported having confidence in their colleagues’ ability to use PAP. Over half (56%) agreed that work is assigned to those with skills appropriate to PAP. One fourth (26%) agreed that sufficient training is provided to enable staff and managers to implement PAP. Respondents in Gothenburg agreed to a greater extent than those in regional areas that work is assigned to those with skills appropriate to PAP (Mdn 4 vs. Mdn 3, *U* = 1116, *p* = 0.032), that sufficient training to implement PAP is provided (Mdn 3 vs. Mdn 2, *U* = 704, *p* < 0.001), and that sufficient resources to support PAP are available (Mdn 3 vs. Mdn 3, *U* = 1121, *p* = 0.029). No differences were seen related to work experience or profession. One fourth (26.9%) reported that sufficient resources are available to support PAP and half (51.1%) agreed that management adequately supports PAP. Option B responses were selected by 1–22 respondents (0.9% to 19.5%).

Barriers from the open-ended questions were inadequate education and insufficient time to use PAP. Statements like “I would like to learn more about PAP, but I have too many duties to have time to plunge into it. It's not my most prioritised task, instead it's something I do on the side, a few times a month” were typical. Facilitators were staff taking on the role of using PAP and having more time with patients when delivering PAP.

### Reflexive monitoring

Thirty-seven percent reported being aware of reports about the effects of PAP. Managers agreed to a higher extent than “other” professions that they were aware of reports (Mdn 4 vs. Mdn 3, *U* = 45, *p* = 0.013) and respondents with more than 10 years of work experience in the organisation agreed to a higher extent than those with 6–10 years of experience that they were aware of reports (Mdn 4 vs. Mdn 2, *U* = 30, *p* = 0.007). Sixty percent agreed that PAP is worthwhile and 48.5% valued the effects PAP has had on their work. Respondents in the Gothenburg area agreed to a greater extent than those in regional areas that they valued the effects (Mdn 4 vs. Mdn 3, *U* = 890, *p* = 0.011). The respondents agreed that feedback about PAP can be used to improve it in the future (81.2%). No differences were seen related to work experience. Option B responses were selected by 2–22 respondents (1.7% to 12.1%).

A reported barrier for using PAP was the lack of research on PAP for children. Statements like “I’d like to see randomised studies that are large enough to show the effectiveness of PAP if I am to become positive about the intervention” are illustrative. The opportunity to provide discounted activities was reported as an important facilitator.

### Acceptability

Most respondents stated that PAP meets with their approval (85.6%), is appealing (85.6%), and that they like (84.0%) and welcome (83.2%) PAP (Table 6). Respondents in the Gothenburg area agreed to a greater extent than those in regional areas that PAP meets their approval (Mdn 5 vs. Mdn 4, *U* = 1528, *p* = 0.022). Respondents with more than 10 years of work experience agreed to a higher extent than those with 2–5 years of experience that they welcome working with PAP (Mdn 5 vs. Mdn 4, *U* = 23, *p* = 0.019). No differences were found by profession.

**Table 6:** Acceptability, appropriateness and feasibility of physical activity on prescription.

Statement	*N* (missing)	Agree *n* (%)	Neutral *n* (%)	Disagree *n* (%)	Median^a^ (IQR)	Mean (SD)^a^
**Acceptability**						
PAP meets my approval	125	107 (85.6)	17 (13.6)	1 (0.8)	5 (5–5)	4.36 (0.75)
PAP is appealing to me	124 (1)	107 (85.6)	16 (12.8)	1 (0.8)	4.5 (4.5–5)	4.35 (0.74)
I like PAP	125	105 (84.0)	18 (14.4)	2 (1.6)	4 (4–5)	4,31 (0.78)
I welcome PAP	124 (1)	104 (83.2)	18 (14.4)	2 (1.6)	5 (5–5)	4.35 (0.79)
**Appropriateness**						
PAP seems fitting	123 (2)	102 (81.6)	16 (12.8)	5 (4.0)	5 (5–5)	4.24 (0.90)
PAP seems suitable	123 (2)	104 (83.2)	15 (12.0)	4 (3.2)	4 (4–5)	4.32 (0.81)
PAP seems applicable	121 (4)	100 (80.0)	19 (15.2)	2 (1.6)	5 (5–5)	4.25 (0.78)
PAP seems like a good match	117 (8)	98 (78.4)	12 (9.6)	7 (5.6)	4 (4–5)	4.24 (0.87)
**Feasibility**						
PAP seems implementable	118 (7)	98 (78.4)	16 (12.8)	4 (3.2)	4 (4–5)	4.23 (0.81)
PAP seems possible	122 (3)	110 (88.0)	12 (9.6)	0 (0.0)	5 (5–5)	4.43 (0.67)
PAP seems doable	122 (3)	103 (82.4)	16 (12.8)	3 (2.4)	4 (4–5)	4.25 (0.78)
PAP seems easy to use	117 (8)	89 (71.2)	26 (20.8)	2 (1.6)	4 (4–5)	3.99 (0.77)

IQR, interquartile range; PAP, physical activity on prescription; SD, standard deviation. In the survey the items in appropriateness were formulated as PAP for children with obesity.

^a^ Medians and means are calculated on the original 5-point scales.

### Appropriateness

Most agreed that PAP seems fitting (81.6%), suitable (83.2%), applicable (80%), and like a good match (78.4%) for children with obesity (Table 6). No differences were seen by practice location, profession, or years of work experience.

### Feasibility

Most respondents reported PAP being implementable (78.4%), possible (88%), doable (82.4%), and easy to use (71.2%) (Table 6). No differences were found by practice location, profession, or years of work experience.

## Discussion

This study reports prerequisites and determinants for implementing the PAP intervention for children with obesity amongst healthcare professionals at paediatric clinics in western Sweden. Our findings suggest that those prerequisites are good, and that, in fact, implementation is underway to various extents. Main findings are that most respondents perceive PAP as familiar and many, in particular nurses and physiotherapists, as a normalised part of their work. Barriers and facilitators for working with PAP were identified across all NPT domains, especially related to collective action and reflexive monitoring. The respondents perceived PAP as highly acceptable, appropriate, and feasible, regardless of profession and experience of working in the organisation.

Respondents from the Gothenburg area perceived PAP as more normalised than those in regional areas; a geographical difference seen in all the NPT domains as well as regarding acceptability of the intervention. Identified facilitators for PAP use were comprehension of the PAP intervention, taking on the role of using PAP, and the interventions's ease of use. Barriers were inadequate education, insufficient time, uncoordinated pathways to other healthcare units, poor collaboration with activity organisers, and the lack of research on PAP for children.

The geographical differences are likely attributed to the PAP support structure that has been in place in Gothenburg for several years. Gothenburg represents a unique context in Sweden, with a PAP support structure in the form of education, networking, and PAP clinics to which patients are referred for extra support in changing their physical activity patterns. None of these support structures are established elsewhere in the region or in Sweden, and there are considerable regional variations across Sweden in the support for work with PAP (37).

Nurses and physiotherapists perceived PAP as normalised to a great extent. Both professions have worked with PAP for many years in Sweden, particularly for adults. Studies in adult populations have also shown nurses' engagement in PAP and other types of physical activity referrals (33, 35, 36). In paediatric health care, nurses have a central role in the work with children and families, including counselling about physical activity and following up intervention effects.

Most respondents perceived PAP as acceptable, appropriate and feasible for children with obesity. Feasibility of PAP as part of an internet-based intervention for children with obesity was recently reported in another Swedish study (29). However, as PAP was one of three intervention components, it is not possible to attribute the results to PAP alone. Amongst adults, feasibility and effects of PAP have recently been shown in two studies, of which one showed sustained results five years after the intervention (50, 51). Although not yet evaluated as a stand-alone intervention in children with obesity, the high acceptability, appropriateness and feasibility of PAP found in our and other studies are important prerequisites for future studies on effectiveness in this population.

Both staff and managers perceived PAP as a possible intervention, implying an understanding of the feasibility of using it in routine clinical practice and the possibility of implementing it in paediatric health care. The high acceptability of PAP by managers is an important prerequisite to the normalisation of PAP. This finding is in contrast to previous studies on PAP, which have identified lack of supportive management (35) and organisational support (33, 35, 36, 38) as problematic.

One reason for the high scores on appropriateness of PAP may be the intervention's person-centredness and individually tailored components, which correspond well with a respectful and structured obesity management according to Swedish national guidelines (32). Another reason might be the discounts offered for many of the prescribed physical activities, which can enable the child's participation in an activity. Families with obese children are often socio-economically disadvantaged (52), so this financial incentive could be an important facilitator.

The collective and individual understanding of an intervention and how it differs from usual ways of working is important for clinical practice (42). In the domain coherence, almost two thirds of the respondents reported they could “make sense” of PAP and understand how it affected their work. These findings were nuanced by qualitative data where respondents expressed insufficient knowledge of PAP and uncertainty about its clinical use. Similar findings have been shown in previous research on PAP for adults, where lack of information and knowledge about PAP and its application was found amongst practitioners (34, 35).

Patients have described not receiving sufficient information about PAP during an intervention period (53). Our findings show a variation in the respondents' perceptions of PAP and its usability in paediatric health care. It is natural for healthcare professionals to experience uncertainty regarding the rationale and clinical use of PAP, particularly in a context for which the intervention has not primarily been developed. This variation in perceptions might reflect that the work with PAP has been transferred from an adult context to the paediatric context without having been fully developed and adapted for children with obesity, which may contribute to uncertainty about its application.

For successful integration into practice, the collective contribution to enact and sustain the work with a new intervention is important. Regardless of profession and years of working in the organisation, most items in the cognitive participation domain were scored high amongst the respondents in our study. In the open-ended questions, respondents described how PAP work was organised in their own clinic and amongst other clinics with licensed practitioners. In the Gothenburg area, key people were driving the PAP work forward and could share good experiences with new colleagues.

The lack of physiotherapists in the paediatric healthcare organisation was described as a barrier, implying that physiotherapists are viewed as one of the most legitimate professions for working with PAP. Physiotherapists' familiarity with PAP and their perception that PAP is already a normal part of their work also corroborate this view. Physiotherapist is a profession with skills for working with physical activity (54), but that is largely missing in paediatric health care. To access these skills and competency, some staff referred patients onward to physiotherapists in PAP clinics or rehabilitation clinics. This uncoordinated referral system between prescribers and physiotherapists was seen as a barrier for working with PAP. Nevertheless, another study described a similar referral setup in primary and secondary care for adults, in which patients perceived PAP to be both feasible and increasing physical activity (50). Hence, the need for formal and coordinated referral pathways between clinics may be greater for children and their families than for adults. The lack of coordination between clinics has been identified earlier as a considerable barrier for families (38).

To improve work with PAP, many respondents called for more training. In the collective action domain, lack of training, structure, and time was described as barriers to efficiently delivering PAP. Similar barriers have also been reported for adult populations managed in primary care (33, 35, 36), as well as for children with intellectual developmental disorders (38). A recent systematic review of implementation of obesity prevention interventions for children also identified lack of knowledge, e.g., concerning physical activity recommendations, as a barrier amongst primary care nurses and physicians (55).

Only half of our respondents, including managers, agreed that management adequately supports PAP. However, in view of the high acceptability and feasibility of PAP reported by both staff and managers, the perceived lack of management support may imply poor communication between staff and managers rather than an actual lack of support. Improved communication and collaboration amongst staff and managers would likely improve chances for an intervention to become normalised in routine practice. Insufficient training, managerial support, and resources were reported as important barriers for implementing physical activity prevention interventions for children with obesity also in primary care (55).

In the reflexive monitoring domain almost 40% of the respondents agreed they were aware of reports about the effects of PAP. This finding is difficult to interpret since research is mostly lacking on PAP for children, but communal or individual evaluations may have been undertaken in clinical practice. Managers reported being aware of effects to a greater extent than other professions, possibly implying they might be better informed by policy documents and national guideline recommendations (30, 56) about the health benefits of physical activity for children. Although lack of research on PAP for children was reported as a barrier, staff might also recognise PAP as an evidence-based intervention for adults and could have gained knowledge through networking, education, and information material for both adults and children.

### Strengths and limitations

A main strength of the study is the use of a theory-based framework and instrument to assess and categorise the factors that might influence implementation of PAP in the paediatric context. A particular strength in using the NPT is its focus on the implementation work healthcare professionals actually do, rather than their cognitions, e.g., beliefs and attitudes. Another strength is our use of validated instruments, which are also pragmatic and easy to use. The NoMAD was particularly helpful in pointing out problems that can be addressed when implementing PAP for children with obesity, enabling improvements related to collective action and reflexive monitoring. Assessing the dual perspective of practitioners and managers also strengthens the findings. Supplementing NoMAD with the AIM, IAM, and FIM instruments to assess important implementation determinants provided a comprehensive overview of aspects necessary to address in a future implementation of PAP for children with obesity. Several efforts were made to reduce bias. Sampling bias was minimised since the survey was distributed to all staff and managers at all paediatric clinics in the study population. We attempted to reduce non-response bias by sending several reminders to answer the questionnaire.

There were some limitations to the study. The intention to capture multiple perspectives meant that not all participants had practical experience of PAP, making several questions irrelevant for some respondents and likely contributing to both unit-level and item-level missing data. The use of self-reported data entails a risk for both self-selection bias and social desirability bias. We did not perform sensitivity analyses, but believe our analyses are robust enough with the used tests. We did not investigate gender, because a vast majority of both practitioners and managers in the population studied are women. The low alpha values for some of the NoMAD items indicate low internal consistency, which might have affected the results of the statistical analyses.

There is an obvious need for research on effectiveness of physical activity promoting interventions for childhood obesity, as well as implementation process and outcome evaluations of such interventions. To improve the understanding of barriers and facilitators for using PAP, further research is needed from the perspective of staff and managers, as well as that of the children and their parents.

Our study can provide helpful information to develop support structures for PAP work, streamline the use of the intervention, and inform future implementation strategies. The broad inclusion criteria of the study, including all professions and managers involved in paediatric health care, and the study setting – Region Västra Götaland which is Sweden's second largest county council – enhances generalisability of our findings to other paediatric populations and to other regions in Sweden, and possibly also to other countries with similar paediatric healthcare systems.

## Conclusions

The prerequisites for implementing PAP for children with obesity in paediatric health care in western Sweden can be considered good. The intervention is familiar and perceived as acceptable, appropriate, and feasible by paediatric healthcare practitioners and managers, constituting important facilitators for implementing PAP. For many participants, PAP was already perceived as a normal part of their work, and a majority believed it would become a normal part of their work in the future. The wide acceptance demonstrates receptiveness to PAP as an intervention to promote an active lifestyle for children with obesity. Barriers and facilitators for working with PAP exist in all NPT domains, particularly in the domains collective action and reflexive monitoring where main barriers are the lack of education, resources, and research on PAP for children.

## Data Availability

The raw data supporting the conclusions of this article will be made available by the authors, without undue reservation.

## References

[1] Garrido-Miguel M Cavero-Redondo I Alvarez-Bueno C Rodriguez-Artalejo F Moreno LA Ruiz JR Prevalence and trends of overweight and obesity in European children from 1999 to 2016: a systematic review and meta-analysis. JAMA Pediatr. (2019) 173(10):e192430. 10.1001/jamapediatrics.2019.2430 31381031 PMC6686782

[2] NCD Risk Factor Collaboration. Worldwide trends in body-mass index, underweight, overweight, and obesity from 1975 to 2016: a pooled analysis of 2416 population-based measurement studies in 128.9 million children, adolescents, and adults. Lancet. (2017) 390(10113):2627–42. 10.1016/S0140-6736(17)32129-3 29029897 PMC5735219

[3] World Health Organization. Global Health Observatory (2020). . Available at: https://www.who.int/data/gho/data/themes/theme-details/GHO/body-mass-index-(bmi) (Accessed October 24, 2022).

[4] Public Health Agency of Sweden. Overweight and obesity are common and increase with age in 6–9 year olds. Report no. 20100. Available at: https://www.folkhalsomyndigheten.se/publicerat-material/publikationsarkiv/oe/overvikt-och-fetma-ar-vanligt-och-okar-med-alder-hos-6-9-aringar/ (Accessed October 24, 2022).

[5] Jenssen BP Kelly MK Powell M Bouchelle Z Mayne SL Fiks AG. COVID-19 and changes in child obesity. Pediatrics. (2021) 147(5):1–3. 10.1542/peds.2021-050123 33653879

[6] Jia P Zhang L Yu W Yu B Liu M Zhang D Impact of COVID-19 lockdown on activity patterns and weight status among youths in China: the COVID-19 impact on lifestyle change survey (COINLICS). Int J Obes (Lond). (2021) 45(3):695–9. 10.1038/s41366-020-00710-4 33277588 PMC7715639

[7] Lange SJ Kompaniyets L Freedman DS Kraus EM Porter R Blanck HM Longitudinal trends in body mass index before and during the COVID-19 pandemic among persons aged 2–19 years—United States, 2018–2020. MMWR Morb Mortal Wkly Rep. (2021) 70(37):1278–83. 10.15585/mmwr.mm7037a3 34529635 PMC8445379

[8] Vogel M Geserick M Gausche R Beger C Poulain T Meigen C Age- and weight group-specific weight gain patterns in children and adolescents during the 15 years before and during the COVID-19 pandemic. Int J Obes (Lond). (2022) 46(1):144–52. 10.1038/s41366-021-00968-2 34556774 PMC8458556

[9] Woolford SJ Sidell M Li X Else V Young DR Resnicow K Changes in body mass index among children and adolescents during the COVID-19 pandemic. JAMA. (2021) 326(14):1434–6. 10.1001/jama.2021.15036 34448817 PMC8511973

[10] Pietrobelli A Pecoraro L Ferruzzi A Heo M Faith M Zoller T Effects of COVID-19 lockdown on lifestyle behaviors in children with obesity living in verona, Italy: a longitudinal study. Obesity (Silver Spring). (2020) 28(8):1382–5. 10.1002/oby.22861 32352652 PMC7267384

[11] Kopelman P Jebb SA Butland B. Executive summary: foresight “tackling obesities: future choices” project. Obes Rev. (2007) 8(Suppl 1):vi–ix. 10.1111/j.1467-789X.2007.00344.x 17316292

[12] Horesh A Tsur AM Bardugo A Twig G. Adolescent and childhood obesity and excess morbidity and mortality in young adulthood-a systematic review. Curr Obes Rep. (2021) 10(3):301–10. 10.1007/s13679-021-00439-9 33950400

[13] Jebeile H Cardel MI Kyle TK Jastreboff AM. Addressing psychosocial health in the treatment and care of adolescents with obesity. Obesity (Silver Spring). (2021) 29(9):1413–22. 10.1002/oby.23194 34431234

[14] Pulgaron ER. Childhood obesity: a review of increased risk for physical and psychological comorbidities. Clin Ther. (2013) 35(1):A18–32. 10.1016/j.clinthera.2012.12.014 23328273 PMC3645868

[15] Simmonds M Llewellyn A Owen CG Woolacott N. Predicting adult obesity from childhood obesity: a systematic review and meta-analysis. Obes Rev. (2016) 17(2):95–107. 10.1111/obr.12334 26696565

[16] Luttikhuis H O Baur L Jansen H Shrewsbury VA O'Malley C Stolk RP Interventions for treating obesity in children. Cochrane Database Syst Rev. (2009) (1):CD001872. 10.1002/14651858.CD001872.pub2 19160202

[17] LeCroy MN Kim RS Stevens J Hanna DB Isasi CR. Identifying key determinants of childhood obesity: a narrative review of machine learning studies. Child Obes. (2021) 17(3):153–9. 10.1089/chi.2020.0324 33661719 PMC8418446

[18] Headid RJ III Park SY. The impacts of exercise on pediatric obesity. Clin Exp Pediatr. (2021) 64(5):196–207. 10.3345/cep.2020.00997 32777917 PMC8103043

[19] Whooten R Kerem L Stanley T. Physical activity in adolescents and children and relationship to metabolic health. Curr Opin Endocrinol Diabetes Obes. (2019) 26(1):25–31. 10.1097/MED.0000000000000455 30507695 PMC6522241

[20] Metcalf B Henley W Wilkin T. Effectiveness of intervention on physical activity of children: systematic review and meta-analysis of controlled trials with objectively measured outcomes (EarlyBird 54). Br Med J. (2012) 345:e5888. 10.1136/bmj.e5888 23044984

[21] Nooijen CF Galanti MR Engstrom K Moller J Forsell Y. Effectiveness of interventions on physical activity in overweight or obese children: a systematic review and meta-analysis including studies with objectively measured outcomes. Obes Rev. (2017) 18(2):195–213. 10.1111/obr.12487 28067022

[22] McGoey T Root Z Bruner MW Law B. Evaluation of physical activity interventions in children via the reach, efficacy/effectiveness, adoption, implementation, and maintenance (RE-AIM) framework: a systematic review of randomized and non-randomized trials. Prev Med. (2016) 82:8–19. 10.1016/j.ypmed.2015.11.004 26582207

[23] Wolfenden L Barnes C Jones J Finch M Wyse RJ Kingsland M Strategies to improve the implementation of healthy eating, physical activity and obesity prevention policies, practices or programmes within childcare services. Cochrane Database Syst Rev. (2020) 2(2):Cd011779. 10.1002/14651858.CD011779.pub3 32036618 PMC7008062

[24] Arsenijevic J Groot W. Physical activity on prescription schemes (PARS): do programme characteristics influence effectiveness? Results of a systematic review and meta-analyses. BMJ Open. (2017) 7(2):e012156. 10.1136/bmjopen-2016-012156 28153931 PMC5293992

[25] Swedish agency for health technology assessment and assessment of social services. Metods of promoting physical activity: a systematic review. *Stockholm: Swedish agency for health technology assessment and assessment of social services (SBU)*. (2006), SBU-rapport:181.

[26] Kallings LV. The Swedish approach on physical activity on prescription. (“Implementation of physical activity in health care—facilitators and barriers”). Clinical Health Promotion. (2016) 2016(6(Supplement)):31–3. Available at: http://www.clinhp.org/ifile/Vol6_Supplement2_HEPA_p31_p33.pdf (Accessed January 5, 2023)

[27] Onerup A Arvidsson D Blomqvist A Daxberg EL Jivegard L Jonsdottir IH Physical activity on prescription in accordance with the Swedish model increases physical activity: a systematic review. Br J Sports Med. (2019) 53(6):383–8. 10.1136/bjsports-2018-099598 30413421

[28] Lauruschkus K Hallstrom I Westbom L Tornberg A Nordmark E. Participation in physical activities for children with cerebral palsy: feasibility and effectiveness of physical activity on prescription. Arch Physiother. (2017) 7:13. 10.1186/s40945-017-0041-9 29340207 PMC5759903

[29] Thorén A Janson A Englund E Silfverdal SA. Development, implementation and early results of a 12-week web-based intervention targeting 51 children age 5–13 years and their families. Obes Sci Pract. (2020) 6(5):516–23. 10.1002/osp4.440 33082993 PMC7556424

[30] The Swedish National Board of Health and Welfare. National guidelines for the prevention and treatment of unhealthy lifestyle habits. Stockholm: The Swedish National Board of Health and Welfare (2021). 21099.

[31] Delisle Nyström C Larsson C Alexandrou C Ehrenblad B Eriksson U Friberg M Results from Sweden's 2018 Report card on physical activity for children and youth. J Phys Act Health. (2018) 15(S2):S413–s4. 10.1123/jpah.2018-0519 30475149

[32] The National Board of Health and Welfare, Sweden. National guidelines for obesity care; support for governance and management. Stockholm: The national board of Health and Welfare (2022). 2022-4-7822.

[33] Bohman DM Mattsson L Borglin G. Primary healthcare nurses’ experiences of physical activity referrals: an interview study. Prim Health Care Res Dev. (2015) 16(3):270–80. 10.1017/S1463423614000267 25075720

[34] Curbach J Apfelbacher C Knoll A Herrmann S Szagun B Loss J. Physicians’ perspectives on implementing the prevention scheme “physical activity on prescription": results of a survey in bavaria. Z Evid Fortbild Qual Gesundhwes. (2018) 131–132:66–72. 10.1016/j.zefq.2018.02.001 29486976

[35] Gustavsson C Nordqvist M Broms K Jerden L Kallings LV Wallin L. What is required to facilitate implementation of Swedish physical activity on prescription?—interview study with primary healthcare staff and management. BMC Health Serv Res. (2018) 18(1):196. 10.1186/s12913-018-3021-1 29562922 PMC5863486

[36] Persson G Brorsson A Ekvall Hansson E Troein M Strandberg EL. Physical activity on prescription (PAP) from the general practitioner's Perspective—a qualitative study. BMC Fam Pract. (2013) 14:128. 10.1186/1471-2296-14-128 23987804 PMC3765882

[37] The Public Health Authority. PAP In Sweden—a description of the regional work with physical activity on prescription (PAP) as a method of treatment. Sweden: The Public Health Authority (2022). 21232.

[38] Boman C Bernhardsson S. Exploring needs, barriers, and facilitators for promoting physical activity for children with intellectual developmental disorders: a qualitative focus group study. J Intellect Disabil. (2022):17446295211064368. 10.1177/17446295211064368 35128986 PMC9940129

[39] May C Finch T. Implementing, embedding, and integration practices: an outline of normalization process theory. Sociology. (2009) 43(3):535–54. 10.1177/0038038509103208

[40] Nilsen P. Making sense of implementation theories, models and frameworks. Implement Sci. (2015) 10:53. 10.1186/s13012-015-0242-0 25895742 PMC4406164

[41] Rycroft-Malone J Bucknall T. Using theory and frameworks to facilitate the implementation of evidence into practice. Worldviews Evid Based Nurs. (2010) 7(2):57–8. 10.1111/j.1741-6787.2010.00194.x 20492634

[42] Rapley T Girling M Mair FS Murray E Treweek S McColl E Improving the normalization of complex interventions: part 1—development of the NoMAD instrument for assessing implementation work based on normalization process theory (NPT). BMC Med Res Methodol. (2018) 18(1):133. 10.1186/s12874-018-0590-y 30442093 PMC6238361

[43] May C. Towards a general theory of implementation. Implement Sci. (2013) 8:18. 10.1186/1748-5908-8-18 23406398 PMC3602092

[44] Proctor E Silmere H Raghavan R Hovmand P Aarons G Bunger A Outcomes for implementation research: conceptual distinctions, measurement challenges, and research agenda. Adm Policy Ment Health. (2011) 38(2):65–76. 10.1007/s10488-010-0319-7 20957426 PMC3068522

[45] Egger M von Elm E Altman DG Pocock SJ Gøtzsche PC Vandenbroucke JP. The strengthening the reporting of observational studies in epidemiology (STROBE) statement: guidelines for reporting observational studies. J Clin Epidemiol. (2008) 61:344e9. 10.1016/j.jclinepi.2007.11.008 18313558

[46] Finch TL Girling M May CR Mair FS Murray E Treweek S Improving the normalization of complex interventions: part 2—validation of the NoMAD instrument for assessing implementation work based on normalization process theory (NPT). BMC Med Res Methodol. (2018) 18(1):135. 10.1186/s12874-018-0591-x 30442094 PMC6238372

[47] Elf M Nordmark S Lyhagen J Lindberg I Finch T Aberg AC. The Swedish version of the normalization process theory measure S-NoMAD: translation, adaptation, and pilot testing. Implement Sci. (2018) 13(1):146. 10.1186/s13012-018-0835-5 30509289 PMC6278165

[48] Weiner BJ Lewis CC Stanick C Powell BJ Dorsey CN Clary AS Psychometric assessment of three newly developed implementation outcome measures. Implement Sci. (2017) 12(1):108. 10.1186/s13012-017-0635-3 28851459 PMC5576104

[49] Bernhardsson S Boman C Lundqvist S Arvidsson D Borjesson M Larsson MEH Implementation of physical activity on prescription for children with obesity in paediatric health care (IMPA): protocol for a feasibility and evaluation study using quantitative and qualitative methods. Pilot Feasibility Stud. (2022) 8(1):117. 10.1186/s40814-022-01075-3 35650617 PMC9158137

[50] Andersen P Holmberg S Årestedt K Lendahls L Nilsen P. Factors associated with increased physical activity among patients prescribed physical activity in Swedish routine health care including an offer of counselor support: a 1-year follow-up. BMC Public Health. (2022) 22(1):509. 10.1186/s12889-022-12940-4 35292017 PMC8925134

[51] Lundqvist S Cider A Larsson MEH Hagberg L Bjork MP Borjesson M. The effects of a 5-year physical activity on prescription (PAP) intervention in patients with metabolic risk factors. PLoS One. (2022) 17(10):e0276868. 10.1371/journal.pone.0276868 36315564 PMC9621409

[52] Olstad DL Teychenne M Minaker LM Taber DR Raine KD Nykiforuk CI Can policy ameliorate socioeconomic inequities in obesity and obesity-related behaviours? A systematic review of the impact of universal policies on adults and children. Obes Rev. (2016) 17(12):1198–217. 10.1111/obr.12457 27484468

[53] Joelsson M Bernhardsson S Larsson ME. Patients with chronic pain may need extra support when prescribed physical activity in primary care: a qualitative study. Scand J Prim Health Care. (2017) 35(1):64–74. 10.1080/02813432.2017.1288815 28277047 PMC5361421

[54] Albert FA Crowe MJ Malau-Aduli AEO Malau-Aduli BS. Functionality of physical activity referral schemes (PARS): a systematic review. Front Public Health. (2020) 8:257. 10.3389/fpubh.2020.00257 32671011 PMC7329989

[55] Ray D Sniehotta F McColl E Ells L. Barriers and facilitators to implementing practices for prevention of childhood obesity in primary care: a mixed methods systematic review. Obes Rev. (2022) 23(4):e13417. 10.1111/obr.13417 35064723 PMC9285925

[56] The Public Health Authority. Guidelines for physical activity and sedentary life: Knowledge support for the promotion of physical activity and reduced sedentary behavior. Sweden: The Public Health Authority (2021). 21099.

